# Intraorbital and Intracranial Complications of Acute Rhinosinusitis: A Rare Case Report

**Published:** 2018-09

**Authors:** Pradeep Pradhan, Dillip-Kumar Samal, Chappity Preetam, Pradipta-Kumar Parida

**Affiliations:** 1 *Department of Otorhinolaryngology, All India Institute of Medical Sciences, Bhubaneswar ,Odisha ,India*

**Keywords:** Foot drop, Sinusitis, Subperiosteal and brain abscess

## Abstract

**Introduction:**

Complications of acute sinusitis affecting multiple sites are very uncommon in the antibiotic era. However, a significant proportion of patients (5–40%) suffering from acute sinusitis can have these complications mostly due to the delayed diagnosis of the disease. Patients can have variable presentations according to the site and extent of the infection.

**Case Report:**

A 21-year-old male student presented with subperiosteal abscess and a brain abscess with a history of acute sinusitis. The patient had short history of left-side hemiplegia with foot drop. Endoscopic orbital decompression was performed and the subperiosteal abscess was drained when it did not respond to medical treatment. Complete clinical and radiological recovery was achieved after 1 month of medical treatment.

**Conclusion:**

Complications affecting the multiple sites in acute sinusitis is very uncommon in the antibiotic era. A proper history and thorough clinical examination along with a radiological evaluation are key factors in the final diagnosis of the patients with suspected complications. A quick multidisciplinary approach among otorhinologsts, ophthalmologists and general physicians is always necessary to avoid unwanted life-threatening complications.

## Introduction

Orbital involvement is the most common complication of sinusitis (accounting for 80% of all complications) because of its close anatomical relationship to the paranasal sinuses. If intraorbital complications are not treated in time, they can progress to life-threatening complications such as optic neuritis, cavernous sinus thrombophlebitis or intracranial complications (1,2). Later complications are comparatively rare in the antibiotic era; however, a significant proportion of patients (5–40%) can be affected by sinusitis, mostly due to the delayed diagnosis of the disease (3). Among paranasal sinuses, frontal sinusitis is the most common predisposing pathology leading to brain abscess where the frontal lobe is mostly affected because of its proximal location. Parietal abscess can be found in patients suffering from sphenoid sinusitis, and temporal lobe abscess is very rare. Sinusitis is presumed to be the underlying cause of 10% of all intracranial abscesses (4). Patients in the adolescent age group get frequently affected by unwanted complications.

Based upon the anatomical sites and the degree of involvement, the patient can have various presentations in complicated sinusitis. Along with a proper history and clinical examination, extensive radiological evaluation (computed tomography [CT] scan/magnetic resonance imaging [MRI] of the paranasal sinus and brain) is always mandated in patients with suspected complications. Conservative treatment is offered as the first line of treatment for all complicated sinusitis. Endoscopic/open surgical drainage is warranted in progressive disease which does not respond to medical treatment. 

We report an adolescent male patient presenting with acute bilateral pan sinusitis with subperiosteal abscess (right side) and a subdural empyema in the right parietal lobe along with left hemiplegia. Orbital complications were managed with endoscopic drainage, although extended medical treatment was needed for complete resolution of the parietal abscess.

## Case Report

**Fig 1:**
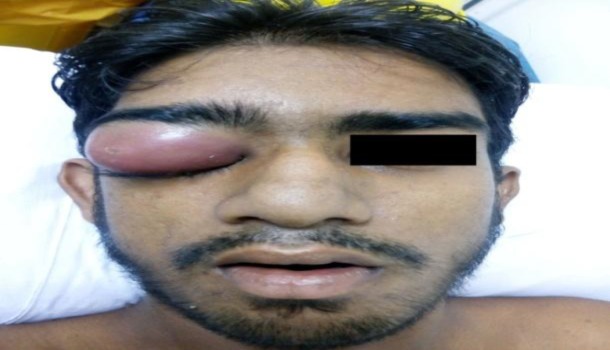
Patient showing ptosis of the right eye with lid abscess

A 21-year-old male student presented to the emergency department with severe headache for 15 days, periorbital swelling, pain and double vision in the right eye for 10 days, fever for 7 days and left lower limb weakness for 5 days (Fig 1).

**Fig 2:**
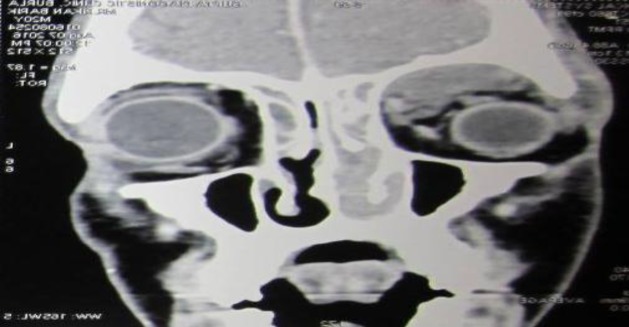
Right subperiosteal abscess pushing the eye ball downward

His past history was unremarkable, and no significant family history was found. The patient was conscious, oriented, febrile, and his vital parameters were normal. Severe ptosis was noticed in the right eye, although visual acuity was normal in both eyes. Fundoscopy was also normal in both eyes. All cranial nerve function was found to be normal. On examination of the lower limb, it was evident that the tone of the muscle was normal, with grade-4 muscle strength. The patient had a typical hemiplegic gait with foot drop due to the weakness of the flexor muscles of the leg and foot on the left side. Ocular movement was restricted on right lateral gaze. The patient’s eye lid was swollen, tender and fluctuant on palpation. Diagnostic nasal endoscopy (DNE) revealed bulging of the right lateral wall of the nose and mucopurulent discharge in the middle meatus. The oral cavity and oropharynx were normal, and examination of the neck revealed no palpable lymph node. Contrast enhanced computer tomography (CECT) of the nose and paranasal sinus demonstrated bilateral pan sinusitis with right subperiosteal abscess (Fig2).

**Fig 3:**
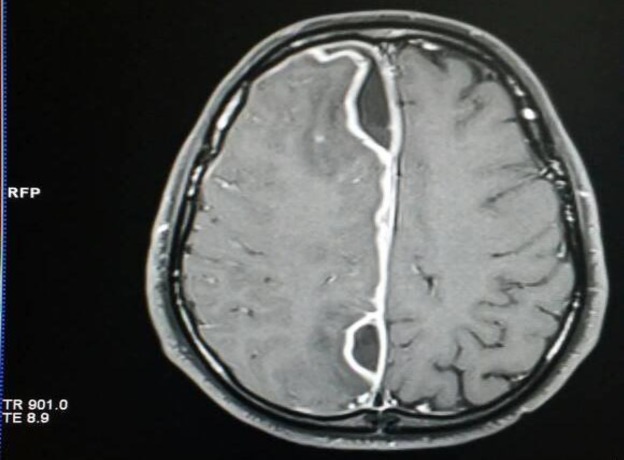
Magnetic resonance image (MRI) of the brain revealing a ring-enhancement lesion in the parasagittal area of right frontal lobe

An MRI of the brain revealed ring-enhancement lesion in the parasagittal area of right frontal lobe without significant displacement of the midline (Fig.3).

Prompt conservative treatment was started covering gram positive, negative and anaerobes, assuming an infective etiology. Vancomycin (I/V 500 mg twice daily), metronidazole (I/V, 500mg thrice daily) and ceftriaxone (I/V 2g twice daily) was given along with supportive treatment (I/V mannitol and dexamethasone). 

After 48 h of treatment, there was no significant improvement in the orbital and intracranial symptoms. Endoscopic orbital decompression was undertaken after 48 h, when it did not respond to medical management. The subperiosteal abscess was drained by breaking the lamina papyracea and was sent for microbiological study for antibiotic sensitivity.

Pus culture was consistent with alpha hemolytic streptococcus, which was sensitive to imipenem and later was continued. 

**Fig 4:**
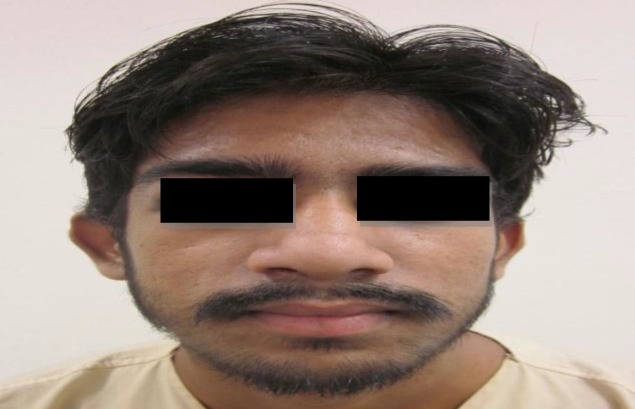
Postoperative photograph showing complete resolution of the subperiosteal abscess

**Fig 5:**
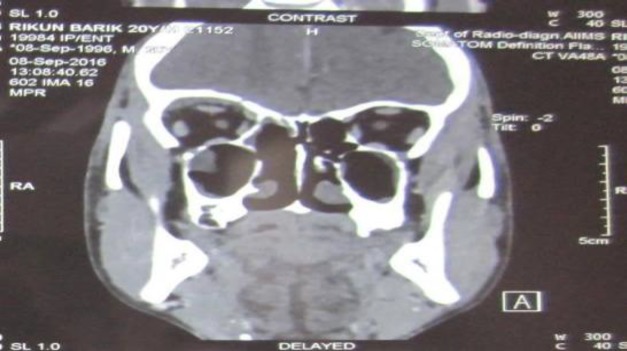
Coronal scan (CT) showing complete clearance of the disease

The fever subsided after 48 h of surgery, and neurological symptoms began resolving after 7 days. The patient subsequently underwent regular physiotherapy for foot drop. A repeat CT scan was advised after 2 weeks, and revealed a reduction in the size of the abscess. The patient was discharged after 28 days of medical treatment with complete resolution of the intracranial abscess and foot drop. The patient remained on close follow-up for last 12 months in the rhinology clinic and was found to be completely asymptomatic, both clinically and radiologically (Figs 4,5).

## Discussion

Although the incidence of complicated sinusitis has been declined recently with the wide use of broad spectrum antibiotics, it is still associated with well-known complications in routine clinical practice, including intraorbital or intracranial abscesses (5). Orbital complications are more frequent, because of the close anatomical relationship between the orbital content and the paranasal sinuses. Further, the multiple blood vessels traversing through the lamina papyracea help in the spread of infections from the paranasal sinuses to the orbit. Again, because of retrograde thrombophlebitis or dehiscent/erosion of lamina, infection from the sinuses can spread to the orbit (6). In contrast, intracranial complications are very rare, and the incidence is about 4% in patients affected with acute or chronic sinusitis (7–9). This is due to retrograde thrombophlebitis or because of the direct spread of the disease from the osteomyelitic bones of the paranasal sinuses mostly to the posterior wall of the frontal sinus. Further, the communicating vessels from the sinuses to the intracranial cavity are valve less, so infection of the sinus mucosa can traverse easily to the intracerebral veins (10). Complications of acute/chronic sinusitis are frequently seen in the adolescent age group, as was documented in our case. A thorough history and clinical examination of the patient is an important step for each case of suspected acute/chronic sinusitis. Although impact on a single site can be seen, it is always necessary to rule out multiple-site involvement, and extensive radiological investigation including both CT scan and MRI is often advised.

Medical treatment is always the first line of treatment offered to patients with complicated sinusitis, irrespective of the site and the severity. Surgery is indicated whenever the condition does not respond to conservative management. Due to the advancement of rigid endoscopes, orbital complications are managed effectively by an intranasal endoscopic approach. Again, most of the intracranial lesions are successfully managed by medical treatment, although this requires long-term drug therapy (4–8 weeks; or even longer as was noticed in our case (11). 

Metronidazole and third-generation cephalosporin (ceftriaxone) are always advised for coverage of anaerobes and gram-negative aerobes, respectively although selective antibiotics can be given based on the microbial culture. Patients should undergo routine ear, nose and throat (ENT) and neurological evaluation in the postoperative period, and a CT scan is advised to confirm disease clearance. Although the response to treatment varies, young patients respond better to conservative treatment (12), as was shown in our case. There is no previous literature demonstrating foot drop as a complication of acute sinusitis, as was noticed in our case. This could be due to the effect on the prefrontal motor cortex, resulting in lower limb weakness. The primary source of infection could be the frontal sinus, because of retrograde thrombophlebitis. Although intraorbital or intracranial complications can be seen in patients with complicated sinusitis, multiple sites can be involved, leading to a life-threatening condition. Vigilance with respect to symptoms over acute or chronic sinusitis and early medical management can reduce unwanted complications. A multidisciplinary approach among otorhinologsts, ophthalmologists and general physicians is always necessary to avoid complications of sinusitis.

## Conclusion

Although multiple complications of acute sinusitis are very uncommon, they can be seen in acute sinusitis patients with variable clinical presentations, according to the site and extent of infection. A proper history and thorough clinical examination along with extensive radiological evaluation is mandatory in patients with suspected complications in order to rule out multiple life-threatening conditions. A rapid multidisciplinary approach among otorhinologsts, ophthalmologists and general physicians is always necessary to improve clinical outcomes.

## References

[1] Sow AJ, Jahendran J, Toh CJ, Kew TY (2012). Sphenoethmoid sinusitis in a child resulting in a disastrous intracranial sequela. Ear, Nose and Throat Journal..

[2] Ketenci İ, Ünlü Y, Vural A, Dogan H, Sahin MI, Tuncer E (2013). Approaches to subperiosteal orbital abscesses. Eur Arch Otorhinolaryngol.

[3] Jones NS, Walker JL, Bassi S, Jones T, Punt J (2002). The intracranial complications of rhinosinusitis: can they be prevented?. Laryngoscope.

[4] Giannoni CM, Stewart MG, Alford EL (1997). Intracranial complications of sinusitis. Laryngoscope.

[5] Kayhan FT, Sayin I, Yazici ZM, Erdur O (2010). Management of orbital subperiosteal abscess. J Craniofac Surg.

[6] Younis RT, Lazar RH, Anand VK (2002). Intracranial complications of sinusitis: a 15-year review of 39 cases. Ear Nose Throat J.

[7] Adnan SB (2000). Instructive case. A swollen eye. J Paediatr Child Health.

[8] Rosen D, Ardekian L, Abu El-Naaj I (2000). Orbital infection arising from a primary tooth: a case report. Int J Paediatr Dent.

[9] Clayman GL, Adams GL, Paugh DR (1991). Intracranial complications of paranasal sinusitis: a combined institutional review. Laryngoscope.

[10] Singh B (1995). The management of sinogenic orbital complications. J Laryngol Otol.

[11] Lu CH, Chang WN, Lui CC (2006). Strategies for the management of bacterial brain abscess. J Clin Neurosci.

[12] Brown CL, Graham SM, Griffin MC, Smith RJ, Carter KD, Nerad JA (2004). Pediatric medial subperiosteal orbital abscess: medical management where possible. Amer J Rhinol.

